# The radical scavenging activity of glycozolidol in physiological environments: a quantum chemical study†

**DOI:** 10.1039/d2ra05907j

**Published:** 2022-11-17

**Authors:** Le Trung Hieu, Mai Van Bay, Nguyen Thi Hoa, Adam Mechler, Quan V. Vo

**Affiliations:** University of Sciences, Hue University Thua Thien Hue 530000 Vietnam; The University of Danang, University of Science and Education Danang 550000 Vietnam; The University of Danang, University of Technology and Education Danang 550000 Vietnam vvquan@ute.udn.vn; Department of Biochemistry and Chemistry, La Trobe University Victoria 3086 Australia

## Abstract

Glycozolidol was isolated from the root of *Glycosmis pentaphylla* (6-hydroxy-2-methoxy-3-methylcarbazole, GLD). This molecule attracted considerable interest due to its beneficial biological activities that likely stem from its antioxidant activity; yet, the radical scavenging action of GLD has not been investigated thus far. In this study, DFT calculations were used to estimate the radical scavenging activity of GLD against a variety of biologically significant radical species in physiological environments. The findings demonstrated that GLD exerts significant antiradical activity in water at pH = 7.40 and in pentyl ethanoate (as a model of lipidic media) with *k*_overall_ = 8.23 × 10^6^ and 3.53 × 10^4^ M^−1^ s^−1^, respectively. In aqueous solution, the sequential proton-loss electron transfer mechanism made the highest contribution to the activity, whereas in nonpolar solvents the formal hydrogen transfer mechanism dominated the activity. GLD is predicted to have strong antiradical activity against CH_3_O˙, CH_3_OO˙, CCl_3_OO˙, NO_2_, SO_4_˙^−^, DPPH and ABTS^+^˙ *k*_app_ ≈ 10^9^ M^−1^ s^−1^ and *k*_f_ ≈ 10^6^ M^−1^ s^−1^. The results suggest that GLD is a good radical scavenger in physiological environments.

## Abstract 2

Glycozolidol (GLD), derived from *Glycosmis pentaphylla*, is predicted to be a good radical scavenger in physiological environments.

## Graphical abstract

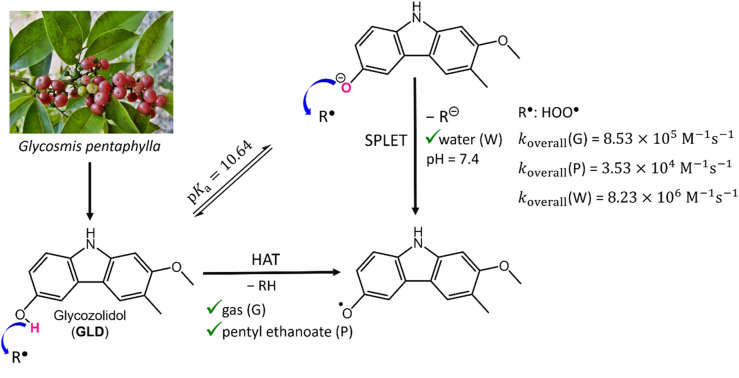

## 1. Introduction

Antioxidants attract considerable interest owing to their roles in important biological processes, and therefore their preferential inclusion in food and pharmaceutical products.^1,2^ They protect against oxidative deterioration in the body and thus against oxidative stress-induced pathological processes such as cancer, aging, hypo sexuality, hyperlipidemia, cardiovascular disease, inflammation, and many others.^1,3,4^

**Fig. 1:**
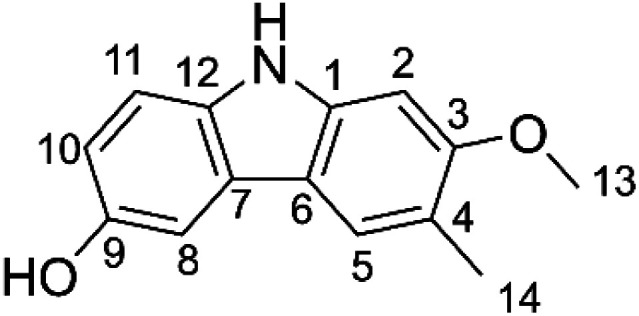
Chemical structure of glycozolidol (GLD).

It is estimated that more than 10 000 individual phytochemicals have been identified in plants, including polysaccharides, phenolics, triterpenoids, steroids, carotenoids, vitamins, essential oils, and alkaloids. Among the great structural diversity of phytochemicals, alkaloid components have attracted considerable attention for known or suspected activity in treating various diseases.^4,5^ Thus, highly substituted carbazole alkaloids are active against *e.g.* neurodegenerative diseases, cancer, tuberculosis and Human Immunodeficiency Virus infections.^6,7^ Among these compounds, glycozolidol (6-hydroxy-2-methoxy-3-methylcarbazole) (Fig. 1) was isolated from the roots of *Glycosmis pentaphylla* and since confirmed to exert antibacterial (against both Gram-positive and Gram-negative bacteria) and antifungal activities.^8,9^ Glycozolidol belongs to a class of natural aromatic nitrogen heterocyclic alkaloids. Based on the oxygenation pattern of tricyclic carbazole alkaloids, glycozolidol could provide robust antioxidant activity due to the presence of amine and quinone groups, however, there are no reports on the mechanism and/or kinetics of the radical scavenging activity of glycozolidol. Herein, we explore the effects of solvent environments and molecular structures on the antioxidant activity and oxidation resistance of glycozolidol against a range of free radicals by using thermodynamic and kinetic calculations.

## 2. Computational details

All density functional theory (DFT) computations in this work were performed with the Gaussian 16 suite of programs^10^ using the M06-2X functional and the 6-311++G(d,p) basis set.^11^ The M06-2X functional is one of the most reliable methods for computing the thermodynamics and kinetics of radical reactions.^12,13^ This method is often used to evaluate the radical scavenging activity of natural and synthesized compounds^12,14–17^ due to its reliable predictions when compared to experimental data.^18–22^ The solvent effects of water and pentyl ethanoate were predicted using the SMD technique,^23^ following a well-established practice in modelling the radical scavenging activity of antioxidants.^12,21,24^

The proton affinity (PA), bond dissociation enthalpy (BDE), and ionization energy (IE) values were calculated as follows.^21^

1 PA = H(GLD^−^) + H(H^+^) − H(GLD–H)

2 BDE = H(GLD˙) + H(H˙) − H(GLD–H)

3 IE = H(GLD–H^+^˙) + H(e^−^) − H(GLD–H)

where H(e), H(H^+^), H(H˙), H(GLD–H^+^˙), H(GLD^−^), H(GLD˙), and H(GLD–H) are the relative enthalpies of the electron, proton, hydrogen atom, cation-radical, anion, radical and neutral molecule, respectively.

The quantum mechanics-based test for overall free radical scavenging activity (QM-ORSA) protocol^20^ was used to complete the kinetic calculations. The rate constants (*k*) were calculated using conventional transition state theory (TST) at 1 M standard state, 298.15 K, following eqn (4).^21,25–31^4
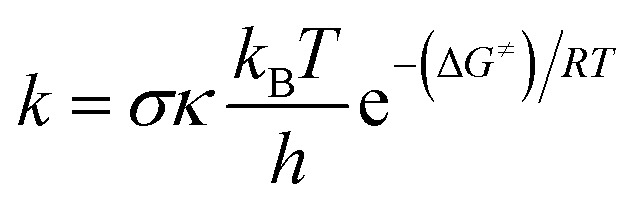
where *s* is the reaction symmetry number,^32,33^*k* stands for tunneling corrections that were calculated using Eckart barrier,^34^*k*_B_ is the Boltzmann constant, *h* is the Planck constant, Δ*G*^≠^ is Gibbs free energy of activation.

The Marcus theory was used to calculate the reaction barriers of single electron transfer (SET) reactions in media.^35,36^ The eqn (5) and (6) were used to compute the Gibbs free energy change of reaction Δ*G*^≠^ for the SET reaction.5
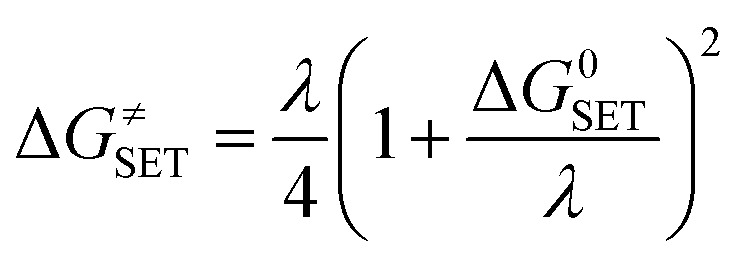

6 *λ* ≈ Δ*E*_SET_ − Δ*G*^0^_SET_

Where ΔG^0^_SET_ represents the conventional Gibbs free energy change of the reaction and Δ*E* represents the nonadiabatic energy difference between reactants and vertical products for SET.^37,38^

For rate constants around the diffusion limit, a modification was made.^20^ Collins–Kimball theory was used to calculate the apparent rate constants (*k*_app_) for an irreversible bimolecular diffusion-controlled reaction in solvents at 298.15 K,^39^ and the literature was used to estimate the steady-state Smoluchowski rate constant (*k*_D_).^20,40^7
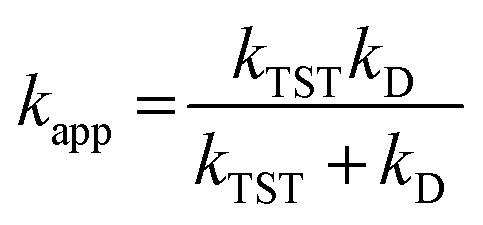

8 *k*_D_ = 4*πR*_AB_*D*_AB_*N*_A_

*D*_AB_ = *D*_A_ + *D*_B_ (denotes the mutual diffusion coefficient of A and B),^39,41^ where *D*_A_ or *D*_B_ is obtained using the Stokes–Einstein formulation eqn (9).^42,43^9
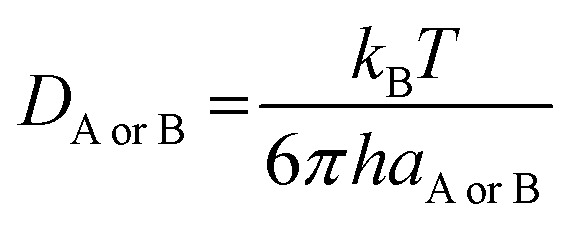


*η* is the viscosity of the solvents (*i.e. η*(pentyl ethanoate) = 8.62 × 10^−4^ Pa s, *η*(H_2_O) = 8.91 × 10^−4^ Pa s) and *a* is the radius of the solute.

The existence of just one imaginary frequency served as a distinguishing feature across all of the transition stages. Intrinsic coordinate calculations were carried out to ensure that each transition state has an accurate connection to both the pre- and post-complexes.

To avoid over-penalizing entropy losses in solution, Okuno's corrections were applied,^44^ which were then updated with the free volume theory utilizing the Benson correction.^20,45–47^

Values for p*K*_a_ are computed in accordance with Galano *et al.* p*K*_a_ = *m*Δ*G*_(BA)_ + *C*_0_, where *m* and *C*_0_ are fitted parameters taken directly from ref. 48 (the M06-2X/6-311++G(d,p) approach), and Δ*G*(BA) is the difference in Gibbs free energy between the conjugated base (B) and the corresponding acid (A).

## 3. Results and discussion

### 3.1 The thermodynamic study

The GLD (GLD–H) can react with free radicals (R˙) *via* either of the three main pathways:

- Formal hydrogen transfer (FHT)^21,49,50^

10 GLD–H + R˙ → GLD˙ + RH

- Sequential electron transfer–proton transfer (SETPT)^51,52^

11 GLD–H → GLD–H^+^˙ + e^−^

12 GLD–H^+^˙ + R^−^ → GLD˙ + RH

- Sequential proton loss electron transfer (SPLET)^52–55^

13 GLD–H → GLD^−^ + H^+^

14 GLD^−^ + R˙ → GLD˙ + RH

In the initial step, the characteristic thermodynamic parameters (BDE, PA, IE) that define the energy barrier of the first step of each mechanism were calculated for all bonds in the HOO˙ radical scavenging activity of GLD in the gas phase (the standard medium for computational chemistry), in pentyl ethanoate for a lipid medium, and in water at pH 7.4 for an aqueous physiological environment. Table 1 provides an overview of the findings.

**Table 1:** The computed thermodynamic parameters (BDE, PA, IE in kcal mol^−1^) of GLD in the studied media (G: the gas phase; P: pentyl ethanoate; W: water)

Positions	BDE			PA			IE		
	G	P	W	G	P	W	G	P	W
C2–H	136.7	116.0	132.0				165.2	122.1	99.2
C5–H	114.4	114.9	115.3						
C8–H	115.6	116.3	129.2						
C10–H	116.6	138.6	116.7						
C11–H	141.9	115.9	132.8						
C13–H	99.9	101.4	101.9						
C14–H	92.7	92.8	92.6						
O9–H	85.2	83.1	82.8	343.6	116.2	84.7			
N–H	91.6	92.5	90.4						

The BDEs ranged from 82.8 to 141.9 kcal mol^−1^, whereas the PA and IE values were in the range of 84.7–343.6 and 99.2–165.2 kcal mol^−1^, respectively. The BDE values varied somewhat randomly, while the PAs and IEs reduced in correlation to the dielectric constant of solvents. The lowest BDE of C–H bond was observed at C14–H (BDE = 92.7, 92.8 and 92.6 kcal mol^−1^ in the gas phase, pentyl ethanoate and water, respectively) that is still higher than BDEs of N–H and O9–H by about 0.3–9.8 kcal mol^−1^. The lowest BDE was found at the O9–H bond (85.2, 83.1 and 82.8 kcal mol^−1^ in the gas phase, pentyl ethanoate and water, respectively). The SPLET and SETPT mechanisms are not favorable in the gas phase or pentyl ethanoate solvent due to the higher PA and IE values compared with the BDE(O9–H). Thus it is expected that the O9–H bond will play a dominant role in the radical scavenging activity of GLD following the FHT reaction in all of the studied media; however, in the aqueous solution, the SPLET mechanism may have a major contribution in the radical scavenging activity if the spontaneous dissociation of the acidic protons eliminates the energy barrier of the first step.

The Gibbs free energies of the GLD + HOO˙ reaction following the proton loss (PL-the first step of SPLET), the FHT, and single electron transfer (SET-the first step of SETPT) mechanisms were calculated in order to eliminate pathways that are not spontaneous thermodynamically, and rate constants were only calculated for the spontaneous reactions. The results are shown in Table 2. The results showed that the process was spontaneous only according to the FHT mechanism, especially at the O9–H bond (Δ*G*^0^ = −7.3 to −3.6 kcal mol^−1^). The Δ*G*^0^ values of the H-abstraction of the N–H bond were 3.0, 3.7 and 0.1 kcal mol^−1^ in the gas phase, pentyl ethanoate and water, respectively, while the lowest Δ*G*^0^ for the C–H bonds was observed at the C14–H bond with 4.1, 4.8 and 1.7 kcal mol^−1^ in the gas phase, pentyl ethanoate and water, respectively. The FHT reactions of the other C–H bonds were not spontaneous (Δ*G*^0^ = 10.6–53.1 kcal mol^−1^). Similarly, neither the proton loss nor SET reactions were spontaneous in any of the investigated solvents. Thus, in nonpolar environments the HOO˙ radical scavenging activity follows the FHT pathway, however in polar media such as water at pH = 7.40, the deprotonation of GLD needs also be addressed.

**Table 2:** The computed Δ*G*^0^ (in kcal mol^−1^) of the HOO˙ + GLD following the formal hydrogen transfer (FHT), proton loss (PL) and single electron transfer (SET) reactions in the studied media (G: the gas phase; P: pentyl ethanoate; W: water)

Positions	FHT			PL			SET		
	G	P	W	G	P	W	G	P	W
C2–H	48.3	25.8	41.3				143.1	60.7	54.3
C5–H	24.4	25.9	23.7						
C8–H	26.5	26.5	38.6						
C10–H	27.2	51.5	25.2						
C11–H	53.1	27.1	42.0						
C13–H	10.9	12.9	10.6						
C14–H	4.1	4.8	1.7						
O9–H	−3.6	−4.3	−7.3	188.4	130.2	89.3			
N–H	3.0	3.7	0.1						

### 3.2 The kinetic study

#### 3.2.1. Acid–base equilibrium

**Fig. 2:**
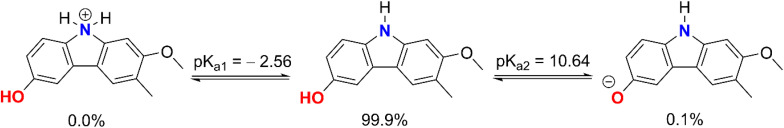
Possible protonation states of GLD at pH = 7.40.

The conjugate base form often exhibits much higher activity than the protonated form of acidic species in aqueous environments.^12,17^ To identify the most probable radical scavenging activity, the protonation state of GLD was initially examined at physiological pH. The GLD structure permits protonation at the N–H and O9–H bonds in accordance with reactions (1) and (2); hence, the p*K*_a_ values of GLD were determined based on the published literature^48^ and are depicted in Fig. 2.

15 R_2_NH_2_^+^ ⇆ R_2_NH + H^+^

16 ROH ⇆ RO^−^ + H^+^

The computed p*K*_a_ values for the amine were p*K*_a1_ = −2.56 (for the cation form of the N–H bond) and p*K*_a2_ = 10.64 (for the O9–H bond). In an aqueous solution with a pH of 7.4, GLD exists in two states: neutral (HA, 99.9%) and anion (A^−^, 0.1%). As a consequence of this, both states were considered during the kinetic analysis of the HOO˙ radical activity of GLD in water at a pH value of 7.4, whereas in nonpolar media (*i.e.* the gas phase and pentyl ethanoate) the neutral state was used to compute the rate constants of the HOO˙ + GLD reaction.

#### 3.2.2. The kinetic study

**Fig. 3:**
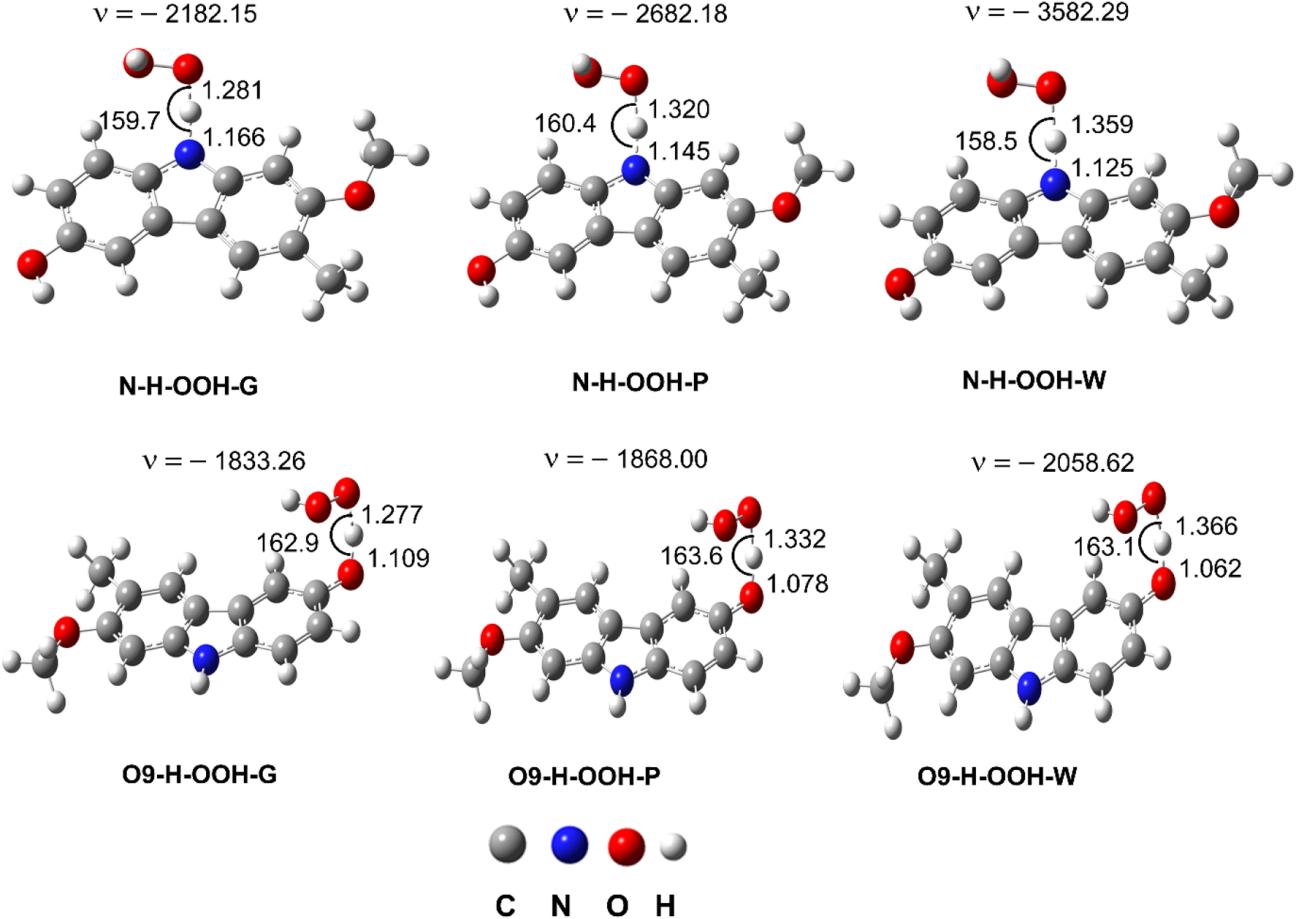
The TS structures of the HOO˙ + GLD reaction following the FHT pathway; imaginary frequencies given in cm^−1^, bond lengths in Å and bond angles in °.

The thermodynamic calculations (Tables 1 and 2) showed that the HOO˙ antiradical activity of GLD in the nonpolar media is dominated by the hydrogen transfer reaction of the N–H and O9–H bonds (Δ*G*^0^ ≈ 0 kcal mol^−1^). The H-abstraction of the C14–H bond (Δ*G*^0^ = 1.7–4.8 kcal mol^−1^) should be omitted due to the lower HOO^·^ radical scavenging activity of C–H bonds compared with the N–H and O9–H bonds.^17,51^ However, in the aqueous solution, the SET reaction of the anion state should be considered.^12,24,50^ Therefore, the total rate constant (*k*_overall_) of GLD antiradical activity against the HOO˙ radical in the gas phase, pentyl ethanoate and water can be calculated using eqn (17) and (18). Table 3 and Fig. 3 show the final results.

**Table 3:** Computed Δ*G*^≠^ in kcal mol^−1^, tunneling correction (*k*), *Γ* in %, and *k*_Eck_, *k*_app_, *k*_f_, and *k*_overall_ in M^−1^ s^−1^ of GLD + HOO˙ reactions

Solv.	Mechanisms		Δ*G*^≠^	*κ*	*k*_Eck_	*k*_app_	*k*_f_ a	*k*_overall_	*Γ*
G	FHT	N–H	15.8	570.5	9.64 × 10^3^			8.53 × 10^5^	1.1
		O9–H	12.0	82.3	8.43 × 10^5^				98.9
P	FHT	N–H	18.8	2915		2.90 × 10^2^		3.53 × 10^4^	0.8
		O9–H	13.8	76.4		3.50 × 10^4^			99.2
W	SET (A^−^)		1.6	15.3^b^		7.90 × 10^9^	7.90 × 10^6^	8.23 × 10^6^	96.0
	FHT	N–H	17.6	4218		3.04 × 10^3^	3.03 × 10^4^		0.0
	(HA)	O9–H	12.8	116.5		3.30 × 10^5^	3.30 × 10^5^		4.0

a *k*_f_ = f.*k*_app_; f(A^−^) = 0.001, f(HA) = 0.999.

b The nuclear reorganization energy (*λ*, in kcal mol^−1^).

Nonpolar environments:

17 *k*_overall_ = *k*_app_(FHT(O9–H)-neutral) + *k*_app_(FHT(N–H)-neutral)

Water at physiological pH:

18 *k*_overall_ = *k*_f_(SET-anion) + *k*_f_(FHT(O9–H)-neutral) + *k*_f_(FHT(N–H)-neutral)

Gas-phase kinetic computations revealed that the HOO˙ antiradical activity of GLD mainly proceeds *via* H-abstraction of the O9–H bond (*k*_(O9–H)_ = 8.43 × 10^5^ M^−1^ s^−1^, *Γ* = 98.9%). The contribution of FHT reactions of the N–H bond was negligible (*Γ* = 1.1%). This is in agreement with thermodynamic data (Table 1). Similar trend was also observed in the lipid medium, H-abstraction at the O9–H bond contributed 99.2% (*k*_(O9–H)_ = 3.50 × 10^4^ M^−1^ s^−1^) to the overall rate constant (*k*_overall_ = 3.53 × 10^4^ M^−1^ s^−1^), whereas, the contribution of the N–H bond was only 0.8% (*k*_(N–H)_ = 2.90 × 10^2^ M^−1^ s^−1^) in the *k*_overall_. In aqueous solution, SPLET was the predominant mechanism with *k*_f_ = 7.90 × 10^6^ M^−1^ s^−1^ (Δ*G*^≠^ = 1.6 kcal mol^−1^), however, the FHT reaction of the O9–H and N–H bond still contributed approximately 4.0% of the *k*_overall_. Thus in the polar environment the HOO˙ antiradical activity of GLD is roughly 233 times faster than in the lipid environment (*k*_overall_ = 8.23 × 10^6^*vs.* 3.53 × 10^4^ M^−1^ s^−1^, respectively). Nevertheless, in the lipid environment the HOO˙ radical scavenging activity of GLD is still higher than that of typical antioxidants such as BHT (*k*_overall_ = 1.70 × 10^4^ M^−1^ s^−1^),^14^ trolox (*k*_overall_ = 3.40 × 10^3^ M^−1^ s^−1^),^19^ ascorbic acid (*k*_overall_ = 5.71 × 10^3^ M^−1^ s^−1^),^20^ or resveratrol (*k*_overall_ = 1.31 × 10^4^ M^−1^ s^−1^).^24^ In the polar environment, GLD is about 33 and 92 times more active than BHT (*k*_overall_ = 2.51 × 10^5^ M^−1^ s^−1^),^14^ and trolox (*k* = 8.96 × 10^4^ M^−1^ s^−1^),^19^ respectively, but less active than ascorbic acid (*k* = 9.97 × 10^7^ M^−1^ s^−1^),^20^ and resveratrol (*k* = 5.62 × 10^7^ M^−1^ s^−1^).^24^ Thus GLD is a promising radical scavenger in physiological environments.

### 3.3 The antiradical activity of GLD against ordinary free radicals in aqueous solution following the SET reaction

Although HOO˙ scavenging activity is a valuable comparative metric, the antiradical activity against different radicals often vary in a broad range. Therefore, the radical scavenging activity of GLD was subsequently modeled against a variety of common free radicals, including HOO˙, CH_3_OO˙, CCl_3_OO˙, HO˙, CH_3_O˙, CCl_3_O˙, NO, NO_2_, O_2_˙^−^, SO_4_˙^−^, N_3_˙, ABTS^+^˙ and DPPH. The hydroperoxyl radical scavenging activity of GLD (*Γ* = 96.0%) is determined by the SET mechanism. Consequently, in this investigation, the antiradical activity against these radicals in water at pH = 7.4 was evaluated using the SET mechanism and the findings are shown in Table 4.

**Table 4:** Calculated kinetic data between GLD–O9-ANION (A^−^) and the selected radicals (Δ*G*^≠^, *λ* in kcal mol^−1^; *k*_D_, *k*_app_ and *k*_f_ in M^−1^ s^−1^)^a^

Radicals	Δ*G*^≠^	*λ*	*k*_D_	*k*_app_	*k*_f_
HO˙	42.5	3.4	8.40 × 10^9^	4.30 × 10^−19^	4.30 × 10^−22^
CH_3_O˙	2.1	4.5	7.90 × 10^9^	7.60 × 10^9^	7.60 × 10^6^
CCl_3_O˙	24.1	21.2	7.50 × 10^9^	1.30 × 10^−5^	1.30 × 10^−8^
HOO˙	1.6	15.3	8.10 × 10^9^	7.90 × 10^9^	7.90 × 10^6^
CH_3_OO˙	2.2	14.7	8.00 × 10^9^	7.60 × 10^9^	7.60 × 10^6^
CCl_3_OO˙	1.4	16.8	7.60 × 10^9^	7.50 × 10^9^	7.50 × 10^6^
NO	71.8	14.3	8.20 × 10^9^	1.50 × 10^−40^	1.50 × 10^−43^
NO_2_	0.0	27.7	8.00 × 10^9^	8.00 × 10^9^	8.00 × 10^6^
O_2_˙^−^	36.8	17.1	8.00 × 10^9^	7.10 × 10^−15^	7.10 × 10^−18^
SO_4_˙^−^	14.1	17.6	7.70 × 10^9^	2.90 × 10^2^	2.90 × 10^−1^
N_3_˙	52.3	2.4	7.90 × 10^9^	3.00 × 10^−26^	3.00 × 10^−29^
DPPH	0.8	18.8	7.40 × 10^9^	7.40 × 10^9^	7.40 × 10^6^
ABTS˙^+^	0.0	11.8	7.40 × 10^9^	7.40 × 10^9^	7.40 × 10^6^

a *k*_f_ = *f*.*k*_app_; *f*(A^−^) = 0.001.

GLD should have high activity against CH_3_O˙, CH_3_OO˙, CCl_3_OO˙, NO_2_, SO_4_˙^−^, DPPH and ABTS^+^˙ radicals with *k*_app_ ≈ 10^9^ M^−1^ s^−1^ and *k*_f_ ≈10^6^ M^−1^ s^−1^, whereas NO, O_2_˙^−^ HO˙, CCl_3_O˙, and N_3_˙ radicals cannot be eliminated by GLD under the studied conditions. By the SET reaction, GLD is less active against CCl_3_O˙, and N_3_˙ radicals than fraxin^55^ or usnic acid,^56^ but more effective against HOO˙ and CH_3_OO˙ radicals. For HO˙, however, the prediction of low activity suggests that SET is not the correct mechanism; highly reactive radicals are known to follow alternative pathways (FHT or radical adduct formation to the neutral species) that for these radicals are essentially barrierless in aqueous media. Thus our results also highlight the limits of generalizations from one detailed study.^50^

## 4. Conclusion

The antiradical activity of GLD against HOO˙, CH_3_OO˙, CCl_3_OO˙, HO˙, CH_3_O˙, CCl_3_O˙, NO, NO_2_, O_2_˙^−^, SO_4_˙^−^, N_3_˙, ABTS˙^+^ and DPPH was studied using DFT calculations. In the physiological environments GLD exhibited significant antiradical activity. The overall rate constants for the antiradical trapping of GLD in water at pH = 7.40 and pentyl ethanoate were *k*_overall_ = 8.23 × 10^6^ and 3.53 × 10^4^ M^−1^ s^−1^, respectively. The SPLET mechanism made contributions to the activity in water at pH 7.40, however, the FHT mechanism characterized the activity in nonpolar solvents. Additionally, it was found that GLD has strong antiradical activity against CH_3_O˙, CH_3_OO˙, CCl_3_OO˙, NO_2_, SO_4_˙^−^, DPPH and ABTS^+^˙ *k*_app_ ≈ 10^9^ M^−1^ s^−1^ and *k*_f_ ≈ 10^6^ M^−1^ s^−1^. According to the computed results, GLD is more effective at trapping HOO^·^ than reference antioxidants like trolox and BHT in the physiological environment. The results suggest that GLD can join the long list of phytochemicals with good radical scavenging activity in physiological environments, emphasizing yet again the importance of varied plant sources in diets and in dietary supplements to maintain health and prevent disease.

## Conflicts of interest

There are no conflicts to declare.

## Supplementary Material

RA-012-D2RA05907J-s001
