# Supplementary material for: The radical scavenging activity of glycozolidol in physiological environments: a quantum chemical study
Source: RSC Adv. 2022 Nov 17;12(50):32693–9. doi: 10.1039/d2ra05907j (PMC9671143; doi:10.1039/d2ra05907j)
Supplement: RA-012-D2RA05907J-s001 [file RA-012-D2RA05907J-s001.pdf]

Electronic Supplementary Material (ESI) for RSC Advances.
This journal is © The Royal Society of Chemistry 2022

Supporting Information (SI)

The radical scavenging activity of glycozolidol in physiological environments: a quantum chemical study

Le Trung Hieu,[1] Mai Van Bay,[2] Nguyen Thi Hoa,[3] Adam Mechler[4] and Quan V. Vo[3*]

[1]University of Sciences, Hue University, Thua Thien Hue 530000, Vietnam
[2]The University of Danang, University of Science and Education, Danang 550000, Vietnam
[3]The University of Danang - University of Technology and Education, Danang 550000, Vietnam.
[4]Department of Chemistry and Physics, La Trobe University, Victoria 3086, Australia.
*Corresponding authors: vvquan@ute.udn.vn

Table of Contents

Table S1: The Cartesian coordinates and energies of TS of the reaction between compound GLD with HOO• following the FHT mechanism in studied environments (G: the gas phase; P: pentyl ethanoate; W: water) S2

Table S1: The Cartesian coordinates and energies of TS of the reaction between compound GLD with HOO• following the FHT mechanism in studied environments (G: the gas phase; P: pentyl ethanoate; W: water)

Name				GLD (gas)
Cartesian Coordinates				Frequency and Energy
O	-4.09745100	-0.20686700	0.00000000	Zero-point correction= 0.242014 (Hartree/Particle)
O	4.87641300	1.52571500	0.00000000	Thermal correction to Energy= 0.256750
N	0.53040800	-1.84774900	0.00000000	Thermal correction to Enthalpy= 0.257694
C	0.00000000	0.35477500	0.00000000	Thermal correction to Gibbs Free Energy= 0.201087
C	1.44114500	0.23277100	0.00000000	Sum of electronic and zero-point Energies= -746.182118
C	-0.51820100	-0.95187700	0.00000000	Sum of electronic and thermal Energies= -746.167382
C	1.72909700	-1.14651900	0.00000000	Sum of electronic and thermal Enthalpies= -746.166438
C	-0.88851500	1.43681500	0.00000000	Sum of electronic and thermal Free Energies= -746.223045
C	-2.25330300	1.22716600	0.00000000	
C	-1.89353600	-1.20475600	0.00000000	
C	-2.74026800	-0.10557500	0.00000000	
C	2.48832400	1.15936900	0.00000000	
C	3.04042400	-1.61505900	0.00000000	
C	3.79169400	0.68997700	0.00000000	
C	4.06683400	-0.68550200	0.00000000	
C	-3.24101700	2.36142000	0.00000000	
C	-4.66443800	-1.49818500	0.00000000	
H	0.44289600	-2.84912900	0.00000000	
H	-0.50963400	2.45387300	0.00000000	
H	-2.26885300	-2.21887600	0.00000000	
H	2.28556000	2.22605900	0.00000000	
H	3.26037000	-2.67614200	0.00000000	
H	5.10299700	-0.99920200	0.00000000	
H	-3.89052000	2.31626900	0.87726500	
H	-2.71968900	3.31912300	0.00000000	
H	-3.89052000	2.31626900	-0.87726500	
H	-5.74277000	-1.35578100	0.00000000	
H	-4.37108600	-2.05818000	-0.89376100	
H	-4.37108600	-2.05818000	0.89376100	
H	4.57834500	2.43844600	0.00000000	
**Name**				**GLD (P)**
Cartesian Coordinates				Frequency and Energy
O	-4.09886600	-0.18094100	0.00000000	Zero-point correction= 0.241861 (Hartree/Particle)
O	4.88555500	1.49280400	0.00000000	Thermal correction to Energy= 0.256473
N	0.51420700	-1.84604700	0.00000000	Thermal correction to Enthalpy= 0.257417
C	0.00101600	0.36219200	0.00000000	Thermal correction to Gibbs Free Energy= 0.201114
C	1.44170200	0.22903800	0.00000000	Sum of electronic and zero-point Energies= -746.204115
C	-0.52551100	-0.94326300	0.00000000	Sum of electronic and thermal Energies= -746.189503
C	1.71599700	-1.15542900	0.00000000	Sum of electronic and thermal Enthalpies= -746.188558
C	-0.88157200	1.45116600	0.00000000	Sum of electronic and thermal Free Energies= -746.244861
C	-2.24817300	1.24777800	0.00000000	
C	-1.90323900	-1.18876000	0.00000000	
C	-2.74438300	-0.08468700	0.00000000	
C	2.49742500	1.14790800	0.00000000	
C	3.02396900	-1.63632500	0.00000000	
C	3.79638100	0.66439900	0.00000000	
C	4.05901000	-0.71480300	0.00000000	
C	-3.22516000	2.39113400	0.00000000	
C	-4.67169700	-1.47638600	0.00000000	
H	0.41799300	-2.84986400	0.00000000	

H	-0.49686700	2.46637500	0.00000000	
H	-2.28178500	-2.20199500	0.00000000	
H	2.30761800	2.21713700	0.00000000	
H	3.23178600	-2.70006600	0.00000000	
H	5.09125500	-1.04400900	0.00000000	
H	-3.87531300	2.35681800	0.87812100	
H	-2.69353900	3.34377400	0.00000000	
H	-3.87531300	2.35681800	-0.87812100	
H	-5.75007500	-1.32956900	0.00000100	
H	-4.38207600	-2.03640400	-0.89437100	
H	-4.38207600	-2.03640300	0.89437100	
H	4.59134900	2.40988900	0.00000000	
**Name**				**GLD (W)**
Cartesian Coordinates				Frequency and Energy
O	-4.10427800	-0.18284100	0.00000000	Zero-point correction= 0.241333 (Hartree/Particle)
O	4.88858200	1.49901200	0.00000000	Thermal correction to Energy= 0.255955
N	0.51097600	-1.84713200	0.00000000	Thermal correction to Enthalpy= 0.256899
C	-0.00086200	0.36335900	0.00000000	Thermal correction to Gibbs Free Energy= 0.200513
C	1.43939600	0.22939900	0.00000000	Sum of electronic and zero-point Energies= -746.203519
C	-0.52714400	-0.94269000	0.00000000	Sum of electronic and thermal Energies= -746.188897
C	1.71181200	-1.15607200	0.00000000	Sum of electronic and thermal Enthalpies= -746.187953
C	-0.88073000	1.45446700	0.00000000	Sum of electronic and thermal Free Energies= -746.244338
C	-2.24815100	1.25063900	0.00000000	
C	-1.90465300	-1.18820400	0.00000000	
C	-2.74176600	-0.08231900	0.00000000	
C	2.49396600	1.14978200	0.00000000	
C	3.01908900	-1.63921900	0.00000000	
C	3.79018400	0.66122800	0.00000000	
C	4.05468700	-0.71728700	0.00000000	
C	-3.22202900	2.39696500	0.00000000	
C	-4.66975500	-1.48816100	0.00000000	
H	0.41267300	-2.85185400	0.00000000	
H	-0.49397400	2.46876700	0.00000000	
H	-2.28292700	-2.20141800	0.00000000	
H	2.31094400	2.21976400	0.00000000	
H	3.22327500	-2.70332600	0.00000000	
H	5.08639700	-1.04864700	0.00000000	
H	-3.87115200	2.36751100	0.87913600	
H	-2.68507000	3.34607900	0.00000000	
H	-3.87115200	2.36751100	-0.87913600	
H	-5.74833800	-1.34732000	0.00000000	
H	-4.37145100	-2.04154200	-0.89402300	
H	-4.37145100	-2.04154200	0.89402300	
H	4.59252200	2.41737200	0.00000000	
**Name**				**GLD-O9-RAD (G)**
Cartesian Coordinates				Frequency and Energy
O	-4.02400100	-0.53942200	0.00000000	Zero-point correction= 0.229746 (Hartree/Particle)
O	4.69737800	1.92716600	0.00000000	Thermal correction to Energy= 0.243849
N	0.74007300	-1.74680100	0.00000000	Thermal correction to Enthalpy= 0.244793
C	0.00000000	0.39715400	0.00000000	Thermal correction to Gibbs Free Energy= 0.188689
C	1.45490700	0.40568800	0.00000000	Sum of electronic and zero-point Energies= -745.554611
C	-0.39907000	-0.94447700	0.00000000	Sum of electronic and thermal Energies= -745.540507
C	1.85895400	-0.96180500	0.00000000	Sum of electronic and thermal Enthalpies= -745.539563
C	-0.97917400	1.39326800	0.00000000	Sum of electronic and thermal Free Energies= -745.595668

C	-2.32100100	1.05664300	0.00000000	
C	-1.73778600	-1.33054000	0.00000000	
C	-2.68519000	-0.31246900	0.00000000	
C	2.40559500	1.39789400	0.00000000	
C	3.21527000	-1.35037100	0.00000000	
C	3.81153800	1.05227900	0.00000000	
C	4.16062300	-0.36609000	0.00000000	
C	-3.40712700	2.09626000	0.00000000	
C	-4.47193900	-1.87807300	0.00000000	
H	0.74046500	-2.75372500	0.00000000	
H	-0.69372200	2.43986600	0.00000000	
H	-2.01583900	-2.37541200	0.00000000	
H	2.15152500	2.45133700	0.00000000	
H	3.48933200	-2.39938400	0.00000000	
H	5.21873600	-0.59746100	0.00000000	
H	-4.04908300	1.99013500	0.87734000	
H	-2.97589600	3.09734300	0.00000000	
H	-4.04908300	1.99013500	-0.87734000	
H	-5.55842400	-1.83305800	0.00000000	
H	-4.12957700	-2.40814200	-0.89433300	
H	-4.12957700	-2.40814200	0.89433300	
**Name**				**GLD-O9-RAD (P)**
Cartesian Coordinates				Frequency and Energy
O	-4.01102900	-0.62082900	0.00000000	Zero-point correction= 0.229809 (Hartree/Particle)
O	4.66079300	2.02204100	0.00000000	Thermal correction to Energy= 0.243834
N	0.77248700	-1.72229400	0.00000000	Thermal correction to Enthalpy= 0.244778
C	-0.01102200	0.40825600	0.00000000	Thermal correction to Gibbs Free Energy= 0.188859
C	1.44372100	0.44388400	0.00000000	Sum of electronic and zero-point Energies= -745.579407
C	-0.38167600	-0.94292300	0.00000000	Sum of electronic and thermal Energies= -745.565383
C	1.87060400	-0.92075900	0.00000000	Sum of electronic and thermal Enthalpies= -745.564438
C	-1.01087000	1.38455100	0.00000000	Sum of electronic and thermal Free Energies= -745.620358
C	-2.34573100	1.01687700	0.00000000	
C	-1.71087100	-1.35950000	0.00000000	
C	-2.68127100	-0.36185400	0.00000000	
C	2.37641900	1.45173300	0.00000000	
C	3.23578100	-1.28448600	0.00000000	
C	3.78717600	1.12843100	0.00000000	
C	4.16178700	-0.28264200	0.00000000	
C	-3.45039300	2.03623500	0.00000000	
C	-4.42529900	-1.97688200	0.00000000	
H	0.79175900	-2.73263700	0.00000000	
H	-0.74904800	2.43767400	0.00000000	
H	-1.96169800	-2.41157300	0.00000000	
H	2.09681800	2.49938700	0.00000000	
H	3.52561100	-2.32921300	0.00000000	
H	5.22315700	-0.50240100	0.00000000	
H	-4.09102600	1.92250800	0.87820900	
H	-3.03606400	3.04503500	0.00000000	
H	-4.09102600	1.92250700	-0.87820900	
H	-5.51327100	-1.95945600	0.00000000	
H	-4.07054000	-2.49695100	-0.89479800	
H	-4.07054000	-2.49695100	0.89479800	
**Name**				**GLD-O9-RAD (H)**
Cartesian Coordinates				Frequency and Energy

O	-4.01402100	-0.62339400	0.00000000	Zero-point correction= 0.229817 (Hartree/Particle)<br>Thermal correction to Energy= 0.243768<br>Thermal correction to Enthalpy= 0.244712<br>Thermal correction to Gibbs Free Energy= 0.188950<br>Sum of electronic and zero-point Energies= -745.579048<br>Sum of electronic and thermal Energies= -745.565096<br>Sum of electronic and thermal Enthalpies= -745.564152<br>Sum of electronic and thermal Free Energies= -745.619915
O	4.66096000	2.02058200	0.00000000	
N	0.77209300	-1.71980900	0.00000000	
C	-0.01368600	0.41272200	0.00000000	
C	1.44064500	0.44632500	0.00000000	
C	-0.38361900	-0.93834000	0.00000000	
C	1.86509200	-0.92197300	0.00000000	
C	-1.01074700	1.39061100	0.00000000	
C	-2.34636400	1.02056700	0.00000000	
C	-1.71004500	-1.35729200	0.00000000	
C	-2.67846400	-0.35786200	0.00000000	
C	2.37267000	1.45163600	0.00000000	
C	3.23095900	-1.28761400	0.00000000	
C	3.77743500	1.11950300	0.00000000	
C	4.15598200	-0.28636200	0.00000000	
C	-3.44875200	2.04225000	0.00000000	
C	-4.41895000	-1.98870500	0.00000000	
H	0.79038700	-2.73222600	0.00000000	
H	-0.74807500	2.44333400	0.00000000	
H	-1.95894600	-2.40962400	0.00000000	
H	2.09186400	2.49902700	0.00000000	
H	3.51734200	-2.33265000	0.00000000	
H	5.21701800	-0.50756500	0.00000000	
H	-4.08866000	1.93221000	0.87927000	
H	-3.02980900	3.04866300	0.00000000	
H	-4.08866000	1.93221000	-0.87927000	
H	-5.50645600	-1.97714700	0.00000000	
H	-4.05622600	-2.50065400	-0.89460800	
H	-4.05622600	-2.50065400	0.89460800	
**Name**				**GLD-N-RAD (G)**
Cartesian Coordinates				Frequency and Energy
O	-4.08519000	-0.20629500	0.00000000	Zero-point correction= 0.228731 (Hartree/Particle)<br>Thermal correction to Energy= 0.242919<br>Thermal correction to Enthalpy= 0.243864<br>Thermal correction to Gibbs Free Energy= 0.187609<br>Sum of electronic and zero-point Energies= -745.544791<br>Sum of electronic and thermal Energies= -745.530603<br>Sum of electronic and thermal Enthalpies= -745.529658<br>Sum of electronic and thermal Free Energies= -745.585913
O	4.91141800	1.37058400	0.00000000	
N	0.51888300	-1.87449300	0.00000000	
C	0.00000000	0.40103600	0.00000000	
C	1.45113800	0.25273400	0.00000000	
C	-0.49365500	-0.92080300	0.00000000	
C	1.66608800	-1.16909200	0.00000000	
C	-0.88004000	1.47106400	0.00000000	
C	-2.25647900	1.24107800	0.00000000	
C	-1.86225300	-1.17958000	0.00000000	
C	-2.73042100	-0.08712700	0.00000000	
C	2.51620800	1.12319700	0.00000000	
C	2.97834400	-1.68455800	0.00000000	
C	3.81415100	0.58030700	0.00000000	
C	4.04174900	-0.80801500	0.00000000	
C	-3.24984300	2.36802500	0.00000000	
C	-4.63314400	-1.50854500	0.00000000	
H	-0.51919000	2.49487900	0.00000000	
H	-2.20993200	-2.20346600	0.00000000	
H	2.37728400	2.20047400	0.00000000	
H	3.13098700	-2.75691100	0.00000000	
H	5.06713600	-1.15416600	0.00000000	
H	-3.89983400	2.31362700	0.87655500	

H	-2.73902900	3.33114000	0.00000000	
H	-3.89983400	2.31362700	-0.87655500	
H	-5.71318400	-1.38081300	0.00000000	
H	-4.33086500	-2.06338100	-0.89332100	
H	-4.33086500	-2.06338100	0.89332100	
H	4.65426600	2.29719000	0.00000000	
**Name**				**GLD-N-RAD (P)**
Cartesian Coordinates				Frequency and Energy
O	-4.08986600	-0.14178400	0.00000000	Zero-point correction= 0.228746 (Hartree/Particle)
O	4.91904300	1.29446000	0.00000000	Thermal correction to Energy= 0.242876
N	0.48892900	-1.88243800	0.00000000	Thermal correction to Enthalpy= 0.243820
C	0.00264500	0.39985800	0.00000000	Thermal correction to Gibbs Free Energy= 0.187628
C	1.45037600	0.22947500	0.00000000	Sum of electronic and zero-point Energies= -745.565515
C	-0.51417300	-0.91259200	0.00000000	Sum of electronic and thermal Energies= -745.551385
C	1.64449400	-1.19891000	0.00000000	Sum of electronic and thermal Enthalpies= -745.550441
C	-0.85966900	1.48671400	0.00000000	Sum of electronic and thermal Free Energies= -745.606633
C	-2.23978200	1.28003200	0.00000000	
C	-1.88649900	-1.14981200	0.00000000	
C	-2.73747400	-0.04138300	0.00000000	
C	2.52574500	1.08572300	0.00000000	
C	2.95335700	-1.73456300	0.00000000	
C	3.81647100	0.52224100	0.00000000	
C	4.02574800	-0.87306300	0.00000000	
C	-3.20877100	2.42755300	0.00000000	
C	-4.66000300	-1.44095900	0.00000000	
H	-0.47975600	2.50369600	0.00000000	
H	-2.25875200	-2.16574500	0.00000000	
H	2.40488800	2.16486400	0.00000000	
H	3.09662700	-2.80894600	0.00000000	
H	5.04548400	-1.23765600	0.00000000	
H	-3.85951700	2.39201000	0.87774900	
H	-2.67543900	3.37888100	0.00000000	
H	-3.85951700	2.39201000	-0.87774900	
H	-5.73837500	-1.29508200	0.00000000	
H	-4.36887600	-1.99937700	-0.89436100	
H	-4.36887600	-1.99937700	0.89436100	
H	4.67562800	2.22890900	0.00000000	
**Name**				**GLD-N-RAD (H)**
Cartesian Coordinates				Frequency and Energy
O	-4.09539300	-0.14198300	0.00000000	Zero-point correction= 0.228602 (Hartree/Particle)
O	4.91183500	1.30267800	0.00000000	Thermal correction to Energy= 0.242712
N	0.48848000	-1.88994300	0.00000000	Thermal correction to Enthalpy= 0.243656
C	0.00057200	0.39317800	0.00000000	Thermal correction to Gibbs Free Energy= 0.187528
C	1.44735900	0.22399900	0.00000000	Sum of electronic and zero-point Energies= -745.566966
C	-0.51714200	-0.91757300	0.00000000	Sum of electronic and thermal Energies= -745.552857
C	1.64295700	-1.20370800	0.00000000	Sum of electronic and thermal Enthalpies= -745.551912
C	-0.85670300	1.48406200	0.00000000	Sum of electronic and thermal Free Energies= -745.608040
C	-2.23761600	1.28096000	0.00000000	
C	-1.88969800	-1.15147100	0.00000000	
C	-2.73504700	-0.03949900	0.00000000	
C	2.51659900	1.08600600	0.00000000	
C	2.95445500	-1.73619600	0.00000000	
C	3.80911200	0.52476000	0.00000000	
C	4.02290600	-0.87134700	0.00000000	

C	-3.20058800	2.43361600	0.00000000	
C	-4.66185100	-1.44830800	0.00000000	
H	-0.47097800	2.49855900	0.00000000	
H	-2.27150200	-2.16388500	0.00000000	
H	2.39654400	2.16445500	0.00000000	
H	3.10565300	-2.80942600	0.00000000	
H	5.04354600	-1.23333600	0.00000000	
H	-3.85008300	2.40424100	0.87891800	
H	-2.65908200	3.37970900	0.00000000	
H	-3.85008300	2.40424100	-0.87891800	
H	-5.74004500	-1.30542400	0.00000000	
H	-4.36430300	-2.00114400	-0.89432000	
H	-4.36430300	-2.00114400	0.89432000	
H	4.66849000	2.23865900	0.00000000	
**Name**				**GLD-O9-HOO-PRE-COMPLEX (G)**
Cartesian Coordinates				Frequency and Energy
O	-4.52821100	0.26563600	0.12484500	Zero-point correction= 0.259503 (Hartree/Particle)
O	4.54378300	0.26501300	-0.93103400	Thermal correction to Energy= 0.277637
N	-0.24989800	-2.13418900	0.31189100	Thermal correction to Enthalpy= 0.278581
C	-0.42531700	0.03886400	-0.30640800	Thermal correction to Gibbs Free Energy= 0.212571
C	0.96876700	-0.33955500	-0.35584600	Sum of electronic and zero-point Energies= -897.071247
C	-1.13854800	-1.09733400	0.11136200	Sum of electronic and thermal Energies= -897.053114
C	1.03532200	-1.69302300	0.03440000	Sum of electronic and thermal Enthalpies= -897.052169
C	-1.12437300	1.22380600	-0.56954600	Sum of electronic and thermal Free Energies= -897.118179
C	-2.49506100	1.27750400	-0.41922400	
C	-2.52713900	-1.08252800	0.27392100	
C	-3.18333800	0.11013500	0.00532300	
C	2.14376900	0.34245600	-0.69959900	
C	2.24972200	-2.37310700	0.09019100	
C	3.35749700	-0.33288900	-0.62282400	
C	3.40548600	-1.68178200	-0.23371900	
C	-3.28455600	2.52919500	-0.68615600	
C	-5.28823700	-0.84280100	0.55514000	
H	-0.49299200	-3.06079500	0.61698200	
H	-0.59077900	2.11219900	-0.89219200	
H	-3.05560900	-1.96951300	0.59498200	
H	2.11577700	1.37194800	-1.04281000	
H	2.29827000	-3.41396900	0.38836100	
H	4.37400300	-2.16344300	-0.18965700	
H	-3.83041100	2.84493100	0.20589200	
H	-2.62214500	3.33795800	-0.99556200	
H	-4.02735700	2.36529500	-1.47026000	
H	-6.32135000	-0.50478200	0.59297600	
H	-5.20329000	-1.67615700	-0.14962600	
H	-4.97529100	-1.17232900	1.55104700	
H	4.46674100	1.21166700	-0.76188700	
O	3.78042400	2.42229300	0.89245800	
O	3.05734100	1.81184100	1.78726500	
H	2.62305200	1.06440300	1.31814000	
**Name**				**GLD-O9-HOO-PRE-COMPLEX (P)**
Cartesian Coordinates				Frequency and Energy
O	-4.53420200	0.23799300	0.15271300	Zero-point correction= 0.258372 (Hartree/Particle)
O	4.52477400	0.34155100	-1.01647500	Thermal correction to Energy= 0.276719
N	-0.22609700	-2.09994900	0.29846100	Thermal correction to Enthalpy= 0.277663

C	-0.43377700	0.07043900	-0.32353300	Thermal correction to Gibbs Free Energy= 0.211245
C	0.96336800	-0.29276500	-0.39195400	Sum of electronic and zero-point Energies= -897.098661
C	-1.12737100	-1.07578400	0.10677300	Sum of electronic and thermal Energies= -897.080314
C	1.04733200	-1.64615400	0.00194900	Sum of electronic and thermal Enthalpies= -897.079370
C	-1.15191100	1.24610700	-0.58414000	Sum of electronic and thermal Free Energies= -897.145789
C	-2.52226700	1.27993500	-0.41875200	
C	-2.51522600	-1.07945200	0.28581800	
C	-3.19110700	0.10309000	0.01929800	
C	2.12788100	0.40372500	-0.74711200	
C	2.27024000	-2.31386800	0.04583100	
C	3.34578900	-0.26462400	-0.69102400	
C	3.41453000	-1.61235100	-0.29792000	
C	-3.32785900	2.52078000	-0.68696700	
C	-5.27596700	-0.89197100	0.57804600	
H	-0.45531200	-3.03291300	0.60570200	
H	-0.63457700	2.14056200	-0.91728300	
H	-3.02601100	-1.97306500	0.61851200	
H	2.08265900	1.43717800	-1.07782900	
H	2.33097700	-3.35273900	0.34922500	
H	4.38615400	-2.09029900	-0.26531700	
H	-3.87180200	2.84058900	0.20566600	
H	-2.67485000	3.33487100	-1.00449400	
H	-4.07282700	2.34936500	-1.46839200	
H	-6.31562400	-0.57275200	0.61919000	
H	-5.17832500	-1.71694600	-0.13425200	
H	-4.95836800	-1.22197300	1.57178000	
H	4.40845700	1.29821100	-0.97776400	
O	3.76129000	2.34689100	1.09463600	
O	3.13832200	1.53705400	1.90400400	
H	2.69873400	0.86899300	1.32912000	
**Name**				**GLD-O9-HOO-PRE-COMPLEX (H)**
Cartesian Coordinates				Frequency and Energy
O	-4.55581500	0.38625300	0.11518100	Zero-point correction= 0.259136 (Hartree/Particle)
O	4.53979700	0.02516700	-0.79860400	Thermal correction to Energy= 0.277177
N	-0.38348400	-2.18611200	0.31492300	Thermal correction to Enthalpy= 0.278121
C	-0.45118900	0.00687500	-0.26470300	Thermal correction to Gibbs Free Energy= 0.212585
C	0.92570200	-0.43138700	-0.29838500	Sum of electronic and zero-point Energies= -897.095805
C	-1.21890000	-1.10942500	0.11823500	Sum of electronic and thermal Energies= -897.077764
C	0.92164800	-1.79418000	0.06887700	Sum of electronic and thermal Enthalpies= -897.076820
C	-1.09519400	1.22827200	-0.50731600	Sum of electronic and thermal Free Energies= -897.142356
C	-2.46670300	1.33374800	-0.37308300	
C	-2.60929700	-1.04246600	0.25788100	
C	-3.20925600	0.18295000	0.00942000	
C	2.13499800	0.20379300	-0.61041400	
C	2.10295800	-2.53133800	0.14095000	
C	3.30843800	-0.53206900	-0.51916400	
C	3.29472500	-1.88729700	-0.15130400	
C	-3.19088400	2.62984100	-0.61236800	
C	-5.36406100	-0.72347700	0.48951600	
H	-0.67418200	-3.11113800	0.59692500	
H	-0.51975000	2.10182500	-0.79802600	
H	-3.17364400	-1.91777400	0.54969400	
H	2.16246600	1.24201800	-0.93033500	
H	2.09282300	-3.57762600	0.42315200	

H 4.23644100 -2.42065500 -0.09793200<br>H -3.72832700 2.95437600 0.28272100<br>H -2.48291800 3.41109100 -0.89068900<br>H -3.93050200 2.53206600 -1.41150300<br>H -6.38542100 -0.35100600 0.52744900<br>H -5.29180500 -1.52321600 -0.25218800<br>H -5.07471000 -1.10234200 1.47299100<br>H 4.45983800 0.98995800 -0.78546300<br>O 4.06865400 2.62720100 0.55068000<br>O 3.39523400 2.15082500 1.55615900<br>H 2.91319800 1.35660900 1.23256500		
**Name**	**GLD-O9-HOO-POST-COMPLEX (G)**	
Cartesian Coordinates	Frequency and Energy	
O -5.00833100 -0.13520700 0.06540300<br>O 3.90066000 1.46468600 -0.12859200<br>N -0.38785200 -1.80586200 -0.08403500<br>C -0.91486600 0.39974900 -0.02321500<br>C 0.53331800 0.26462100 -0.06125600<br>C -1.44431900 -0.89513100 -0.03863200<br>C 0.80065300 -1.13756400 -0.09785100<br>C -1.78902700 1.48754100 0.02307100<br>C -3.15732500 1.28448000 0.05232600<br>C -2.81331600 -1.14829600 -0.01047200<br>C -3.65521500 -0.04137400 0.03455600<br>C 1.57673400 1.15558800 -0.06826900<br>C 2.11175800 -1.65809600 -0.13495400<br>C 2.93547500 0.66663400 -0.11166500<br>C 3.15221200 -0.77589200 -0.14001800<br>C -4.13528100 2.42509800 0.10180400<br>C -5.58815900 -1.42284600 0.05220800<br>H -0.48647400 -2.80803600 -0.10487400<br>H -1.40161600 2.50070600 0.03665300<br>H -3.19392100 -2.16021900 -0.02339100<br>H 1.43218300 2.22916500 -0.04523300<br>H 2.28163500 -2.72849600 -0.15444200<br>H 4.18144500 -1.11519100 -0.14704300<br>H -4.76386600 2.36225100 0.99299900<br>H -3.60792600 3.37897200 0.11101200<br>H -4.80509000 2.40176300 -0.76072800<br>H -6.66432400 -1.26907500 0.07871000<br>H -5.32056900 -1.96643600 -0.85937300<br>H -5.28150600 -2.00213800 0.92887600<br>H 5.53656100 0.84857600 -0.35648400<br>O 6.39029100 0.36585500 -0.39577600<br>O 6.25831900 -0.51649100 0.71440800<br>H 6.80507800 -0.08860900 1.38223600	Zero-point correction= 0.259546 (Hartree/Particle)<br>Thermal correction to Energy= 0.277844<br>Thermal correction to Enthalpy= 0.278789<br>Thermal correction to Gibbs Free Energy= 0.210711<br>Sum of electronic and zero-point Energies= -897.078660<br>Sum of electronic and thermal Energies= -897.060362<br>Sum of electronic and thermal Enthalpies= -897.059417<br>Sum of electronic and thermal Free Energies= -897.127495	
**Name**	**GLD-O9-HOO-POST-COMPLEX (P)**	
Cartesian Coordinates	Frequency and Energy	
O -4.60192900 0.52230300 0.10106400<br>O 4.41126000 -0.20077800 -0.55824900<br>N -0.52770700 -2.21106000 0.24001600<br>C -0.51348800 0.02174700 -0.17508300<br>C 0.85476600 -0.47220300 -0.20742100<br>C -1.33190400 -1.07953600 0.10491300	Zero-point correction= 0.259220 (Hartree/Particle)<br>Thermal correction to Energy= 0.277361<br>Thermal correction to Enthalpy= 0.278305<br>Thermal correction to Gibbs Free Energy= 0.211894<br>Sum of electronic and zero-point Energies= -897.108712<br>Sum of electronic and thermal Energies= -897.090571	

C	0.77313400	-1.87767900	0.05603300	Sum of electronic and thermal Enthalpies= -897.089627
C	-1.10432600	1.27459200	-0.36073900	Sum of electronic and thermal Free Energies= -897.156038
C	-2.47810100	1.41807900	-0.26475200	
C	-2.71626200	-0.98368700	0.21205400	
C	-3.27297900	0.27892200	0.02504900	
C	2.07772200	0.11021400	-0.42078200	
C	1.91717800	-2.70754600	0.09273300	
C	3.27286300	-0.70092900	-0.37973400	
C	3.13137900	-2.12746100	-0.12444000	
C	-3.15375400	2.74614100	-0.45906000	
C	-5.46619300	-0.56736600	0.37939000	
H	-0.86635800	-3.14391100	0.43428100	
H	-0.49360400	2.14309200	-0.58561300	
H	-3.31609100	-1.85671600	0.43105000	
H	2.18344400	1.16835700	-0.63544800	
H	1.81942100	-3.76908700	0.28966300	
H	4.04449400	-2.71056500	-0.10622700	
H	-3.70834400	3.04038800	0.43566000	
H	-2.41715100	3.51921500	-0.68061800	
H	-3.87414200	2.70476600	-1.27990200	
H	-6.47359600	-0.15671800	0.39742100	
H	-5.39978400	-1.33154400	-0.40079600	
H	-5.23824800	-1.01146800	1.35290400	
H	4.54976800	1.50583400	-0.30776200	
O	4.50635800	2.45097000	-0.03498800	
O	4.14965000	2.32551700	1.33858000	
H	3.22121900	2.59196400	1.33504800	
**Name**				**GLD-O9-HOO-POST-COMPLEX (H)**
Cartesian Coordinates				Frequency and Energy
O	-4.60788500	0.52989200	0.07816000	Zero-point correction= 0.258968 (Hartree/Particle)
O	4.41769600	-0.21387500	-0.47474900	Thermal correction to Energy= 0.276906
N	-0.54183200	-2.21913000	0.19640800	Thermal correction to Enthalpy= 0.277850
C	-0.51278500	0.02656400	-0.15719100	Thermal correction to Gibbs Free Energy= 0.212041
C	0.85341900	-0.47302800	-0.17800400	Sum of electronic and zero-point Energies= -897.112757
C	-1.33940600	-1.07898800	0.07597200	Sum of electronic and thermal Energies= -897.094819
C	0.75854700	-1.88602500	0.04915400	Sum of electronic and thermal Enthalpies= -897.093875
C	-1.09309400	1.28759300	-0.30948600	Sum of electronic and thermal Free Energies= -897.159684
C	-2.46933200	1.43251900	-0.22844600	
C	-2.72322700	-0.98375200	0.16451900	
C	-3.27022600	0.28732600	0.01049100	
C	2.08055800	0.10862600	-0.35748100	
C	1.89851900	-2.72209600	0.09330900	
C	3.26418300	-0.71367200	-0.31543100	
C	3.11758300	-2.14118100	-0.08671200	
C	-3.13093900	2.77289000	-0.38396500	
C	-5.47245600	-0.57266500	0.33385500	
H	-0.88948400	-3.15662600	0.35897800	
H	-0.47420100	2.15974300	-0.49302400	
H	-3.32656400	-1.86233900	0.34808700	
H	2.19389300	1.17224300	-0.54076900	
H	1.78966200	-3.78619900	0.26646800	
H	4.02679100	-2.73039100	-0.06361400	
H	-3.69872000	3.03990500	0.51111700	
H	-2.38159600	3.54493700	-0.56018700	

H	-3.83261200	2.77267900	-1.22198100	
H	-6.48022100	-0.16419700	0.35504900	
H	-5.39459300	-1.31999300	-0.45969500	
H	-5.24068100	-1.03012700	1.29900900	
H	4.56015000	1.46173200	-0.36340800	
O	4.58052400	2.41917200	-0.11144100	
O	4.11109600	2.36930100	1.23135300	
H	3.16149200	2.53194300	1.13902700	
**Name**				**GLD-N-HOO-PRE-COMPLEX (G)**
Cartesian Coordinates				Frequency and Energy
O	4.26757300	0.01873500	-0.09033500	Zero-point correction= 0.258883 (Hartree/Particle)
O	-4.72939800	-1.55062500	-0.24997200	Thermal correction to Energy= 0.277402
N	-0.29638700	1.66056300	-0.83177600	Thermal correction to Enthalpy= 0.278346
C	0.17765700	-0.50077500	-0.32003000	Thermal correction to Gibbs Free Energy= 0.211477
C	-1.25758500	-0.35990700	-0.42256300	Sum of electronic and zero-point Energies= -897.069908
C	0.72326800	0.77273800	-0.54815200	Sum of electronic and thermal Energies= -897.051389
C	-1.50850000	0.99944800	-0.70667800	Sum of electronic and thermal Enthalpies= -897.050445
C	1.03495500	-1.57173000	-0.03822400	Sum of electronic and thermal Free Energies= -897.117314
C	2.39904700	-1.37622500	0.02953700	
C	2.09874200	1.00992400	-0.48625000	
C	2.91544000	-0.07201500	-0.19239500	
C	-2.33200800	-1.24760300	-0.26402100	
C	-2.80913300	1.48627900	-0.81789000	
C	-3.62480200	-0.75776700	-0.38584800	
C	-3.86074400	0.60026700	-0.65205500	
C	3.35756200	-2.49217000	0.34065400	
C	4.86170100	1.28686900	-0.26574800	
H	-0.20006400	2.66049200	-0.76716600	
H	0.63213900	-2.56445300	0.13595900	
H	2.49668800	2.00043000	-0.65824500	
H	-2.15580200	-2.29837200	-0.05418900	
H	-3.00133100	2.53234900	-1.02470600	
H	-4.88763500	0.93469300	-0.72599400	
H	3.92768800	-2.27505100	1.24693200	
H	2.81842400	-3.42917700	0.48262900	
H	4.08285400	-2.62382400	-0.46554100	
H	5.93069100	1.14176500	-0.12648200	
H	4.67494200	1.67471800	-1.27229200	
H	4.49066000	2.00110400	0.47609000	
H	-4.45978100	-2.45967300	-0.09671500	
O	-0.77057500	1.46847600	2.22485700	
O	-1.69352900	0.59807100	2.53436000	
H	-1.92092100	0.13980500	1.69885600	
**Name**				**GLD-N-HOO-PRE-COMPLEX (P)**
Cartesian Coordinates				Frequency and Energy
O	4.16709000	-0.33819800	-0.03544200	Zero-point correction= 0.258076 (Hartree/Particle)
O	-4.77289000	-2.23659900	0.10664400	Thermal correction to Energy= 0.276754
N	-0.48538500	1.18836600	-0.28297800	Thermal correction to Enthalpy= 0.277698
C	0.08291100	-0.98997200	-0.00266100	Thermal correction to Gibbs Free Energy= 0.209905
C	-1.36045500	-0.89562900	-0.03307300	Sum of electronic and zero-point Energies= -897.092671
C	0.57466600	0.31963000	-0.16085000	Sum of electronic and thermal Energies= -897.073993
C	-1.66804800	0.47122100	-0.20682800	Sum of electronic and thermal Enthalpies= -897.073049
C	0.99366800	-2.04521000	0.14344900	Sum of electronic and thermal Free Energies= -897.140842
C	2.35449600	-1.80558100	0.13083500	

C	1.94575000	0.60131100	-0.17766700	
C	2.81516100	-0.47043900	-0.03304700	
C	-2.39314900	-1.83424200	0.07552600	
C	-2.98778200	0.91460300	-0.27182600	
C	-3.70348000	-1.38747100	0.00888800	
C	-3.99975500	-0.02599700	-0.16295500	
C	3.36126600	-2.91259600	0.28542900	
C	4.70582200	0.96301300	-0.18657200	
H	-0.40678400	2.19036300	-0.39457100	
H	0.63520000	-3.06253000	0.26743500	
H	2.29702900	1.61722900	-0.29993000	
H	-2.17691600	-2.88994900	0.21073200	
H	-3.22070000	1.96510500	-0.40359600	
H	-5.03979800	0.27499000	-0.20744000	
H	4.00653400	-2.74278300	1.15134400	
H	2.85536000	-3.87068300	0.41339400	
H	4.01428400	-2.98086000	-0.58873200	
H	5.78777000	0.84510100	-0.17528900	
H	4.39978200	1.40934500	-1.13763500	
H	4.40425700	1.61441100	0.63967900	
H	-4.45677400	-3.13851000	0.22601600	
O	-0.07375300	4.24959800	-0.18576600	
O	-0.96843500	4.52183000	0.72225800	
H	-0.84408100	5.46133100	0.96599500	

**Name**				**GLD-N-HOO-PRE-COMPLEX (H)**
Cartesian Coordinates				Frequency and Energy
O	4.24687900	-0.02752000	-0.00433800	Zero-point correction= 0.257901 (Hartree/Particle)
O	-4.54538600	-2.57375700	0.01457200	Thermal correction to Energy= 0.276494
N	-0.50750800	1.17526600	-0.10143800	Thermal correction to Enthalpy= 0.277439
C	0.21499900	-0.97513900	0.01143800	Thermal correction to Gibbs Free Energy= 0.209844
C	-1.23150600	-0.98154000	-0.01051800	Sum of electronic and zero-point Energies= -897.094400
C	0.61074500	0.37498100	-0.05145100	Sum of electronic and thermal Energies= -897.075807
C	-1.63385700	0.37015100	-0.08369100	Sum of electronic and thermal Enthalpies= -897.074863
C	1.19818200	-1.97215300	0.07486700	Sum of electronic and thermal Free Energies= -897.142458
C	2.53938400	-1.63513700	0.07124900	
C	1.95809600	0.75385900	-0.05793900	
C	2.90022900	-0.26203700	0.00061000	
C	-2.19476900	-1.99646200	0.02317800	
C	-2.98122900	0.72548200	-0.12324600	
C	-3.53135100	-1.63480400	-0.01704400	
C	-3.92529000	-0.28962100	-0.09049100	
C	3.61941700	-2.68013300	0.13709100	
C	4.68189000	1.32328800	-0.10644400	
H	-0.50863400	2.18586900	-0.18610500	
H	0.91315100	-3.01854400	0.12607400	
H	2.23362400	1.79854700	-0.10889800	
H	-1.91177600	-3.04312700	0.07918700	
H	-3.28477300	1.76443100	-0.17863300	
H	-4.98364300	-0.05808200	-0.11918200	
H	4.27467600	-2.52085400	0.99744900	
H	3.17696200	-3.67338400	0.22185200	
H	4.25091400	-2.65946800	-0.75536600	
H	5.76921100	1.28802300	-0.11126900	

H 4.32503000 1.77743900 -1.03439300<br>H 4.33850800 1.90957900 0.74976100<br>H -4.16478900 -3.45930100 0.06431600<br>O -0.72275700 4.20457200 -0.28159100<br>O -1.76541100 4.33035100 0.48495600<br>H -1.93215800 5.28842800 0.59083200	
**Name**	**GLD-N-HOO-POST-COMPLEX (G)**
Cartesian Coordinates	Frequency and Energy
O 4.26865200 0.16818700 0.00320900<br>O -4.43088300 -2.59335300 -0.00386200<br>N -0.52152600 1.19337200 -0.07810000<br>C 0.30351600 -0.98035900 0.00550200<br>C -1.15279600 -1.03001400 -0.00955600<br>C 0.61933700 0.39150600 -0.03874900<br>C -1.56321300 0.35070700 -0.05834200<br>C 1.31989900 -1.92272400 0.05058000<br>C 2.65221500 -1.51181200 0.05057900<br>C 1.93783300 0.83374600 -0.04232000<br>C 2.94458100 -0.13293800 0.00223500<br>C -2.09226800 -2.03301600 0.00884900<br>C -2.93600300 0.69325400 -0.08325500<br>C -3.45176100 -1.66638500 -0.01982400<br>C -3.86575800 -0.31907500 -0.06473200<br>C 3.78768900 -2.49433400 0.10097200<br>C 4.63776000 1.53195200 -0.05373200<br>H -0.77821400 2.97330400 -0.29196700<br>H 1.09806700 -2.98470400 0.08660600<br>H 2.14829800 1.89396800 -0.07893500<br>H -1.81258100 -3.08199000 0.04354100<br>H -3.22088000 1.73919400 -0.10542700<br>H -4.92885200 -0.11719100 -0.08196400<br>H 4.42573600 -2.30568300 0.96749000<br>H 3.41042800 -3.51557500 0.15700300<br>H 4.42287900 -2.40178000 -0.78335400<br>H 5.72502600 1.54939100 -0.04710000<br>H 4.27002900 2.00076400 -0.97137900<br>H 4.25835400 2.07923200 0.81452400<br>H -4.05505000 -3.47832200 0.02395500<br>O -1.06997800 3.91334000 -0.34096000<br>O -2.28311500 3.85505700 0.40363000<br>H -2.03613900 4.28690000 1.22832400	Zero-point correction= 0.258533 (Hartree/Particle)<br>Thermal correction to Energy= 0.276949<br>Thermal correction to Enthalpy= 0.277893<br>Thermal correction to Gibbs Free Energy= 0.210396<br>Sum of electronic and zero-point Energies= -897.069394<br>Sum of electronic and thermal Energies= -897.050978<br>Sum of electronic and thermal Enthalpies= -897.050034<br>Sum of electronic and thermal Free Energies= -897.117532
**Name**	**GLD-N-HOO-POST-COMPLEX (P)**
Cartesian Coordinates	Frequency and Energy
O 4.24858700 0.09764800 0.00326200<br>O -4.48177500 -2.53301700 -0.00211500<br>N -0.52580100 1.19643000 -0.09792400<br>C 0.26743100 -0.98796400 0.00530100<br>C -1.18847100 -1.01680400 -0.01263600<br>C 0.60639700 0.37790600 -0.05015800<br>C -1.57851300 0.37132900 -0.07376200<br>C 1.26817100 -1.94800700 0.06482700<br>C 2.60715500 -1.55879800 0.06431400<br>C 1.93171200 0.79979900 -0.05366000<br>C 2.92357600 -0.18399700 0.00289200	Zero-point correction= 0.258055 (Hartree/Particle)<br>Thermal correction to Energy= 0.276421<br>Thermal correction to Enthalpy= 0.277365<br>Thermal correction to Gibbs Free Energy= 0.210247<br>Sum of electronic and zero-point Energies= -897.097162<br>Sum of electronic and thermal Energies= -897.078796<br>Sum of electronic and thermal Enthalpies= -897.077851<br>Sum of electronic and thermal Free Energies= -897.144970

C -2.13845900 -2.00849200 0.01558600<br>C -2.94874400 0.73065600 -0.09937400<br>C -3.49391200 -1.62313900 -0.01954100<br>C -3.89097500 -0.26723200 -0.07354700<br>C 3.72123700 -2.56442400 0.12432600<br>C 4.64028500 1.45932700 -0.08141000<br>H -0.69878900 2.94328500 -0.29679500<br>H 1.02680900 -3.00555400 0.10981500<br>H 2.16426900 1.85577100 -0.09818200<br>H -1.87226000 -3.06019800 0.06242400<br>H -3.22711500 1.77798100 -0.13055300<br>H -4.95101200 -0.04641700 -0.09207300<br>H 4.36558400 -2.38493500 0.98877000<br>H 3.31976300 -3.57613200 0.19296200<br>H 4.35696100 -2.50116700 -0.76296300<br>H 5.72855000 1.45884000 -0.07981800<br>H 4.27828600 1.91606900 -1.00703700<br>H 4.27653600 2.02807500 0.77932000<br>H -4.11798500 -3.42709000 0.03495000<br>O -0.90072200 3.91176300 -0.35566800<br>O -2.06741800 3.99903200 0.45747600<br>H -1.71970200 4.36209900 1.28207100	
**Name**	**GLD-N-HOO-POST-COMPLEX (H)**
Cartesian Coordinates	Frequency and Energy
O 4.19878800 -0.11692700 -0.00256500<br>O -4.65105300 -2.32662400 -0.00643100<br>N -0.52124000 1.21565900 -0.07284200<br>C 0.16426700 -1.00620900 0.01640300<br>C -1.29084200 -0.96240000 -0.00178600<br>C 0.57102800 0.33964200 -0.03039800<br>C -1.61429500 0.44373200 -0.05485100<br>C 1.11482100 -2.01718800 0.06292000<br>C 2.47203600 -1.69451400 0.05928000<br>C 1.91485900 0.69717500 -0.03709500<br>C 2.85388300 -0.33664900 0.00525400<br>C -2.28142400 -1.91189200 0.01485800<br>C -2.96794800 0.86478900 -0.08530400<br>C -3.61846500 -1.46456900 -0.01912400<br>C -3.95312400 -0.08926100 -0.06752000<br>C 3.53022900 -2.75952600 0.10691000<br>C 4.65327100 1.22998100 -0.09507500<br>H -0.35861800 2.90383900 -0.24039200<br>H 0.81803100 -3.06053000 0.10100000<br>H 2.20246200 1.73994200 -0.07522100<br>H -2.07043600 -2.97591000 0.05108400<br>H -3.20757300 1.92123700 -0.12230500<br>H -5.00193300 0.18015100 -0.08949700<br>H 4.19600300 -2.61559700 0.96173800<br>H 3.07191800 -3.74549600 0.18706200<br>H 4.15306000 -2.73830000 -0.79162400<br>H 5.73958900 1.17669500 -0.10849700<br>H 4.29632300 1.69693100 -1.01624200<br>H 4.32564100 1.81171600 0.76994800<br>H -4.34112600 -3.24266300 0.02799100	Zero-point correction= 0.257106 (Hartree/Particle)<br>Thermal correction to Energy= 0.275426<br>Thermal correction to Enthalpy= 0.276370<br>Thermal correction to Gibbs Free Energy= 0.209235<br>Sum of electronic and zero-point Energies= -897.100888<br>Sum of electronic and thermal Energies= -897.082568<br>Sum of electronic and thermal Enthalpies= -897.081624<br>Sum of electronic and thermal Free Energies= -897.148759

O	-0.30827600	3.90212400	-0.29613000		
O	-1.51792600	4.28804500	0.34900100		
H	-1.23669300	4.46676800	1.25700400		
**Name**				**TS-N-H-OOH (G)**	
Cartesian Coordinates				Frequency and Energy	
O	4.24318300	-0.01307800	-0.03857300	Zero-point correction=	0.254280 (Hartree/Particle)
O	-4.62061400	-2.19497700	0.17288000	Thermal correction to Energy=	0.271710
N	-0.45287900	1.26307600	-0.65247100	Thermal correction to Enthalpy=	0.272654
C	0.20201300	-0.86527300	-0.05127200	Thermal correction to Gibbs Free Energy=	0.208575
C	-1.25321200	-0.82183700	-0.09982500	Sum of electronic and zero-point Energies=	-897.047292
C	0.63239300	0.43421500	-0.35887700	Sum of electronic and thermal Energies=	-897.029862
C	-1.58697000	0.51569400	-0.44475300	Sum of electronic and thermal Enthalpies=	-897.028918
C	1.14493300	-1.85021200	0.22651200	Sum of electronic and thermal Free Energies=	-897.092998
C	2.49916800	-1.54021300	0.22173800		
C	1.97989800	0.78403000	-0.37029200		
C	2.90080600	-0.21648500	-0.07084700		
C	-2.25215300	-1.75677200	0.10084700		
C	-2.92149200	0.92004400	-0.56982500		
C	-3.58387600	-1.33951400	-0.01665800		
C	-3.91439800	-0.01717500	-0.34316600		
C	3.55311900	-2.56890700	0.51979200		
C	4.72287000	1.28619000	-0.32099600		
H	-0.44383600	2.36143600	-0.26045600		
H	0.83775900	-2.86611900	0.45342400		
H	2.27512700	1.79826600	-0.60171700		
H	-2.02117400	-2.78933300	0.34542000		
H	-3.16094000	1.94196500	-0.83306900		
H	-4.96223800	0.24183100	-0.42279200		
H	4.14399000	-2.28010300	1.39233700		
H	3.09719300	-3.54090300	0.70960600		
H	4.25103000	-2.66426500	-0.31520300		
H	5.80578300	1.23216000	-0.23545600		
H	4.45154900	1.59374500	-1.33556800		
H	4.33479800	2.01392800	0.39805000		
H	-4.29053600	-3.07123300	0.38900400		
O	-0.51825800	3.34736200	0.55381200		
O	-1.85651800	3.38698800	0.87753800		
H	-1.86928400	3.19401100	1.82418700		
**Name**				**TS-N-H-OOH (P)**	
Cartesian Coordinates				Frequency and Energy	
O	4.24358900	-0.01315100	-0.04664600	Zero-point correction=	0.253516 (Hartree/Particle)
O	-4.61125800	-2.21013000	0.15779800	Thermal correction to Energy=	0.271212
N	-0.45502000	1.26208600	-0.63739500	Thermal correction to Enthalpy=	0.272156
C	0.20268800	-0.86337300	-0.03373700	Thermal correction to Gibbs Free Energy=	0.206789
C	-1.25306900	-0.82421000	-0.09138300	Sum of electronic and zero-point Energies=	-897.073792
C	0.63447600	0.43557300	-0.34162000	Sum of electronic and thermal Energies=	-897.056095
C	-1.58949700	0.51194500	-0.44201600	Sum of electronic and thermal Enthalpies=	-897.055151
C	1.14544200	-1.84992200	0.24326000	Sum of electronic and thermal Free Energies=	-897.120518
C	2.50059800	-1.53988200	0.23412100		
C	1.98187700	0.78536600	-0.36235400		
C	2.90450200	-0.21675700	-0.06598900		
C	-2.24757100	-1.76471400	0.10325200		
C	-2.92478400	0.91057500	-0.58232100		
C	-3.58098000	-1.35186600	-0.02701900		

C	-3.91506700	-0.03096200	-0.36258000	
C	3.54939100	-2.57300800	0.53211300	
C	4.72488900	1.28363200	-0.36378800	
H	-0.44968300	2.34364500	-0.26263400	
H	0.83694800	-2.86536100	0.47118400	
H	2.28132900	1.79644700	-0.60451800	
H	-2.01492700	-2.79545500	0.35379300	
H	-3.17036000	1.92847600	-0.85688900	
H	-4.96254500	0.22825300	-0.45459800	
H	4.14529800	-2.28974600	1.40386200	
H	3.08646000	-3.54120300	0.72640800	
H	4.24398600	-2.68043300	-0.30500600	
H	5.80935000	1.22695400	-0.29222100	
H	4.44314800	1.57017400	-1.38138800	
H	4.35255800	2.02664900	0.34740700	
H	-4.27720000	-3.08683500	0.38169900	
O	-0.52706800	3.37719900	0.55560000	
O	-1.86286800	3.42345600	0.89068000	
H	-1.87574500	3.21046100	1.83558100	
**Name**				**TS-N-H-OOH (W)**
Cartesian Coordinates				Frequency and Energy
O	4.24693900	-0.00952800	-0.04428800	Zero-point correction= 0.253554 (Hartree/Particle)
O	-4.61035400	-2.19663500	0.16459000	Thermal correction to Energy= 0.271264
N	-0.45247300	1.26347200	-0.66190600	Thermal correction to Enthalpy= 0.272208
C	0.20560700	-0.85811200	-0.04926500	Thermal correction to Gibbs Free Energy= 0.206939
C	-1.25012800	-0.81787000	-0.10235400	Sum of electronic and zero-point Energies= -897.077447
C	0.63921400	0.43866600	-0.36200800	Sum of electronic and thermal Energies= -897.059737
C	-1.58685400	0.51832300	-0.45446100	Sum of electronic and thermal Enthalpies= -897.058793
C	1.14595200	-1.84418300	0.23827300	Sum of electronic and thermal Free Energies= -897.124062
C	2.50132500	-1.53542100	0.23301700	
C	1.98648800	0.78839300	-0.37647800	
C	2.90670700	-0.21395000	-0.07085700	
C	-2.24349100	-1.75701600	0.09977900	
C	-2.92332700	0.91956900	-0.58727500	
C	-3.57809800	-1.34265600	-0.02492000	
C	-3.91233900	-0.02038500	-0.36036300	
C	3.54871600	-2.56826400	0.54207800	
C	4.72902700	1.29265200	-0.34121600	
H	-0.44752800	2.33083500	-0.30565200	
H	0.83399700	-2.85667800	0.47234900	
H	2.28925900	1.79821800	-0.61744100	
H	-2.01143200	-2.78666300	0.35087700	
H	-3.16869000	1.93738200	-0.85970600	
H	-4.95983100	0.23905700	-0.44651600	
H	4.15369200	-2.26607000	1.40017500	
H	3.08377400	-3.52898800	0.76210200	
H	4.23305500	-2.69486300	-0.30026300	
H	5.81153000	1.23726500	-0.26133200	
H	4.45095200	1.58965600	-1.35566800	
H	4.34525700	2.02341700	0.37525800	
H	-4.28762200	-3.07768500	0.38509400	
O	-0.54945200	3.37594300	0.55708700	
O	-1.87628500	3.34714200	0.93133000	
H	-1.85866800	3.07694300	1.86062700	

Name				TS-O9-H-OOH (G)
Cartesian Coordinates				Frequency and Energy
				Zero-point correction= 0.254030 (Hartree/Particle)
O	-4.67493900	0.19574400	0.25352900	Thermal correction to Energy= 0.271293
O	4.30523800	0.68229800	-1.03864500	Thermal correction to Enthalpy= 0.272237
N	-0.29164400	-2.01576700	0.08756700	Thermal correction to Gibbs Free Energy= 0.207723
C	-0.57960600	0.20192200	-0.28694300	Sum of electronic and zero-point Energies= -897.052755
C	0.83071000	-0.11038600	-0.41175500	Sum of electronic and thermal Energies= -897.035491
C	-1.23419300	-0.99838400	0.02194200	Sum of electronic and thermal Enthalpies= -897.034547
C	0.95717400	-1.50443400	-0.17056300	Sum of electronic and thermal Free Energies= -897.099062
C	-1.33302100	1.37407400	-0.40649400	
C	-2.70227500	1.34872500	-0.22108600	
C	-2.61371900	-1.06885200	0.21683700	
C	-3.32924500	0.11569600	0.09070600	
C	1.96153300	0.63582700	-0.70318800	
C	2.19489500	-2.15785200	-0.21225300	
C	3.21641300	-0.00474100	-0.74753300	
C	3.31022400	-1.40146900	-0.49139200	
C	-3.55014800	2.58495500	-0.33706100	
C	-5.37762900	-0.98608100	0.57403700	
H	-0.49113400	-2.98098300	0.29113500	
H	-0.84702500	2.31411900	-0.64613700	
H	-3.09446100	-2.00791300	0.45354500	
H	1.91644200	1.69898800	-0.91290900	
H	2.27307700	-3.22171400	-0.02239300	
H	4.29714600	-1.84568200	-0.52568700	
H	-4.07543200	2.78734800	0.59914200	
H	-2.93288400	3.44830800	-0.58629200	
H	-4.31338500	2.46484900	-1.10917900	
H	-6.42390200	-0.70180200	0.65932900	
H	-5.26756400	-1.73668800	-0.21504800	
H	-5.03375000	-1.40185600	1.52645400	
H	4.68728600	1.15956000	-0.11376800	
O	4.81644800	1.54530900	1.09710200	
O	4.01348800	0.68007000	1.77025700	
H	3.15853900	1.13247300	1.83937100	

Name				TS-O9-H-OOH (P)
Cartesian Coordinates				Frequency and Energy
O	-4.65501500	0.22689900	0.23606400	Zero-point correction= 0.253898 (Hartree/Particle)
O	4.34186100	0.56832200	-1.02729400	Thermal correction to Energy= 0.271241
N	-0.30180000	-2.03564300	0.13579300	Thermal correction to Enthalpy= 0.272186
C	-0.55906400	0.17818100	-0.28885100	Thermal correction to Gibbs Free Energy= 0.207660
C	0.84695200	-0.15645900	-0.39995900	Sum of electronic and zero-point Energies= -897.082239
C	-1.22959000	-1.00904400	0.04358600	Sum of electronic and thermal Energies= -897.064896
C	0.94975700	-1.54940300	-0.12805400	Sum of electronic and thermal Enthalpies= -897.063952
C	-1.29772700	1.35661000	-0.44249800	Sum of electronic and thermal Free Energies= -897.128478
C	-2.66892300	1.34992000	-0.26365900	
C	-2.61096000	-1.05941600	0.23463400	
C	-3.31271600	0.13052200	0.07763700	
C	1.99030600	0.56676200	-0.70113000	
C	2.17956600	-2.22193000	-0.15148700	
C	3.23305200	-0.09632100	-0.72224000	
C	3.30743400	-1.48771700	-0.44050500	
C	-3.49626800	2.59507900	-0.41983500	

C	-5.36956600	-0.94516700	0.59075900	
H	-0.51647600	-2.99713800	0.35917200	
H	-0.79974300	2.28492800	-0.70368100	
H	-3.10119000	-1.98847700	0.49175000	
H	1.95589600	1.62701300	-0.93068100	
H	2.23751500	-3.28338300	0.05885800	
H	4.28542600	-1.95364700	-0.46248700	
H	-4.02896600	2.83432800	0.50411300	
H	-2.86052800	3.44170000	-0.68254400	
H	-4.25204500	2.47357800	-1.20015100	
H	-6.41389400	-0.64923600	0.66916400	
H	-5.26962400	-1.71665700	-0.17877200	
H	-5.03028300	-1.33679400	1.55438300	
H	4.62666600	1.17223900	-0.18067700	
O	4.72180400	1.69827800	1.03983900	
O	3.90211800	0.89760000	1.76641600	
H	3.02017700	1.30255400	1.70040700	
**Name**				**TS-O9-H-OOH (W)**
Cartesian Coordinates				Frequency and Energy
O	-4.65171200	0.23645800	0.22713200	Zero-point correction= 0.253912 (Hartree/Particle)
O	4.35322100	0.53306100	-1.03034100	Thermal correction to Energy= 0.271346
N	-0.30548000	-2.04092700	0.15580100	Thermal correction to Enthalpy= 0.272290
C	-0.55319800	0.17116400	-0.28721300	Thermal correction to Gibbs Free Energy= 0.207144
C	0.85096800	-0.17044700	-0.39461100	Sum of electronic and zero-point Energies= -897.0848978
C	-1.22877800	-1.01089500	0.05423800	Sum of electronic and thermal Energies= -897.0674638
C	0.94758200	-1.56173100	-0.10994600	Sum of electronic and thermal Enthalpies= -897.0665198
C	-1.28708600	1.35225400	-0.44754400	Sum of electronic and thermal Free Energies= -897.1316658
C	-2.65867000	1.35057800	-0.27065000	
C	-2.61076500	-1.05617800	0.24226300	
C	-3.30741200	0.13507300	0.07540200	
C	1.99724500	0.54520500	-0.70159200	
C	2.17520700	-2.23997500	-0.12532600	
C	3.23538600	-0.12453600	-0.71332500	
C	3.30623600	-1.51174600	-0.41829700	
C	-3.48346200	2.59786800	-0.43348900	
C	-5.37309100	-0.93500800	0.57573900	
H	-0.52413600	-2.99765100	0.39083200	
H	-0.78574700	2.27741100	-0.71225700	
H	-3.10295400	-1.98209600	0.50490400	
H	1.96794000	1.60373000	-0.93822100	
H	2.22947300	-3.29933500	0.09274900	
H	4.28179200	-1.98197800	-0.43631700	
H	-4.01806700	2.83812400	0.48853400	
H	-2.84656600	3.44217100	-0.69720300	
H	-4.23683100	2.47133400	-1.21458000	
H	-6.41577300	-0.63632800	0.64656100	
H	-5.26330400	-1.70425000	-0.19312300	
H	-5.03763500	-1.32721500	1.53938600	
H	4.62356400	1.16190000	-0.21815200	
O	4.72002500	1.72548000	1.02251600	
O	3.85468900	0.97336100	1.74352400	
H	2.99584300	1.42403400	1.67972500	
